# Bidirectional Mendelian Randomisation Analysis Provides Evidence for the Causal Involvement of Dysregulation of CXCL9, CCL11 and CASP8 in the Pathogenesis of Ulcerative Colitis

**DOI:** 10.1093/ecco-jcc/jjac191

**Published:** 2022-12-28

**Authors:** Jie Chen, Yajing Zhou, Yuhao Sun, Shuai Yuan, Rahul Kalla, Jing Sun, Jianhui Zhao, Lijuan Wang, Xuejie Chen, Xuan Zhou, Siqi Dai, Yu Zhang, Gwo-tzer Ho, Dajing Xia, Qian Cao, Zhanju Liu, Susanna C Larsson, Xiaoyan Wang, Kefeng Ding, Jonas Halfvarson, Xue Li, Evropi Theodoratou, Jack Satsangi

**Affiliations:** Department of Big Data in Health Science, School of Public Health and Second Affiliated Hospital, Zhejiang University School of Medicine, Zhejiang, China; Centre for Global Health, Zhejiang University School of Medicine, Hangzhou, China; Department of Gastroenterology, Third Xiangya Hospital, Central South University, Changsha, China; Department of Big Data in Health Science, School of Public Health and Second Affiliated Hospital, Zhejiang University School of Medicine, Zhejiang, China; Centre for Global Health, Zhejiang University School of Medicine, Hangzhou, China; Department of Big Data in Health Science, School of Public Health and Second Affiliated Hospital, Zhejiang University School of Medicine, Zhejiang, China; Unit of Cardiovascular and Nutritional Epidemiology, Institute of Environmental Medicine, Karolinska Institutet, Stockholm, Sweden; Edinburgh IBD Science Unit, Centre for Inflammation Research, University of Edinburgh, Edinburgh, UK; Department of Big Data in Health Science, School of Public Health and Second Affiliated Hospital, Zhejiang University School of Medicine, Zhejiang, China; Department of Big Data in Health Science, School of Public Health and Second Affiliated Hospital, Zhejiang University School of Medicine, Zhejiang, China; Department of Big Data in Health Science, School of Public Health and Second Affiliated Hospital, Zhejiang University School of Medicine, Zhejiang, China; Department of Gastroenterology, Third Xiangya Hospital, Central South University, Changsha, China; Department of Big Data in Health Science, School of Public Health and Second Affiliated Hospital, Zhejiang University School of Medicine, Zhejiang, China; Colorectal Surgery and Oncology, Key Laboratory of Cancer Prevention and Intervention, Ministry of Education, Second Affiliated Hospital, Zhejiang University School of Medicine, Zhejiang, China; Department of Gastroenterology, Sir Run Run Shaw Hospital, College of Medicine Zhejiang University, Zhejiang, China; Edinburgh IBD Science Unit, Centre for Inflammation Research, University of Edinburgh, Edinburgh, UK; Department of Toxicology of School of Public Health, & Center of Immunology & Infection, Zhejiang University School of Medicine, Zhejiang, China; Department of Gastroenterology, Sir Run Run Shaw Hospital, College of Medicine Zhejiang University, Zhejiang, China; Center for IBD Research, Shanghai Tenth People’s Hospital, Tongji University School of Medicine, Shanghai, China; Unit of Cardiovascular and Nutritional Epidemiology, Institute of Environmental Medicine, Karolinska Institutet, Stockholm, Sweden; Unit of Medical Epidemiology, Department of Surgical Sciences, Uppsala University, Uppsala, Sweden; Department of Gastroenterology, Third Xiangya Hospital, Central South University, Changsha, China; Colorectal Surgery and Oncology, Key Laboratory of Cancer Prevention and Intervention, Ministry of Education, Second Affiliated Hospital, Zhejiang University School of Medicine, Zhejiang, China; Department of Gastroenterology, Faculty of Medicine and Health, Örebro University, Örebro, Sweden; Department of Big Data in Health Science, School of Public Health and Second Affiliated Hospital, Zhejiang University School of Medicine, Zhejiang, China; Centre for Global Health, Usher Institute, University of Edinburgh, Edinburgh, UK; Cancer Research UK Edinburgh Centre, Medical Research Council Institute of Genetics and Cancer, University of Edinburgh, Edinburgh, UK; Translational Gastroenterology Unit, Nuffield Department of Medicine, Experimental Medicine Division, University of Oxford, John Radcliffe Hospital, Oxford, UK

**Keywords:** Systemic inflammatory proteins, ulcerative colitis, Mendelian randomisation

## Abstract

**Background and Aims:**

Systemic inflammation is well recognised to be associated with ulcerative colitis [UC], but whether these effects are causal or consequential remains unclear. We aimed to define potential causal relationship of cytokine dysregulation with different tiers of evidence.

**Methods:**

We first synthesised serum proteomic profiling data from two multicentred observational studies, in which a panel of systemic inflammatory proteins was analysed to examine their associations with UC risk. To further dissect observed associations, we then performed a bidirectional two-sample Mendelian randomisation [TSMR] analysis from both forward and reverse directions using five genome-wide association study [GWAS] summary level data for serum proteomic profiles and the largest GWAS of 28 738 European-ancestry individuals for UC risk.

**Results:**

Pooled analysis of serum proteomic data identified 14 proteins to be associated with the risk of UC. Forward MR analysis using only cis-acting protein quantitative trait loci [cis-pQTLs] or trans-pQTLs further validated causal associations of two chemokines and the increased risk of UC: C-X-C motif chemokine ligand 9 [CXCL9] [OR 1.45, 95% CI 1.08, 1.95, *p = *0.012] and C-C motif chemokine ligand 11 [CCL11] [OR 1.14, 95% CI 1.09, 1.18, *p *= 3.89 × 10^-10^]. Using both cis- and trans-acting pQTLs, an association of caspase-8 [CASP8] [OR 1.04, 95% CI 1.03, 1.05, *p *= 7.63 × 10^-19^] was additionally identified. Reverse MR did not find any influence of genetic predisposition to UC on any of these three inflammation proteins.

**Conclusion:**

Pre-existing elevated levels of CXCL9, CCL11 and CASP8 may play a role in the pathogenesis of UC.

## Graphical Abstract

**Graphical Abstract:**
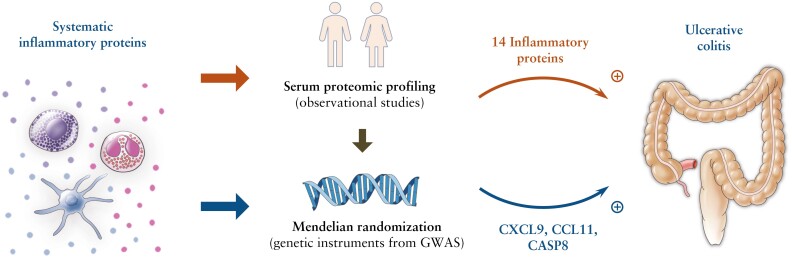

## 1. Introduction

Ulcerative colitis [UC] is a subtype of inflammatory bowel disease [IBD] characterised by chronic, relapsing inflammation of the colon, on the background of a systemic immune response.^1^ The incidence of UC has been increasing worldwide and the prevalence rate is projected to be 1% among Western populations by 2030,^2^ posing a substantial burden on global health.^3,4^

Although the cause of UC remains unclear, systemic inflammation is a hallmark feature of this disease, triggered by immune dysregulation in response to the exposome in a genetically susceptible individual.^5,6^ A wide range of inflammatory cytokines, including interleukin-1 receptor antagonist [IL1R], IL12, IL23, IL17, tumour necrosis factor [TNF] alpha, C-C motif chemokine ligand [CCL] 2 and 3 have been reported to be associated with UC risk in observational studies.^7^ These findings underline the complexity of intestinal inflammation in UC and indicate the possibility of cytokines as potential targets for primary prevention and therapy of UC. Current evidence supports that TNF-mediated, as well as several non-TNF pathways [eg, IL-1, IL-12/23] are involved in disease pathogenesis and represent targets for current and future therapeutic strategies in UC.^8,9^ However, the causal role of specific inflammatory pathways remains unclear, primarily driven by the limitations of observational studies [eg, residual confounding and reverse causality] and the lack of high-quality data from randomised trials. These research gaps and limitations preclude the differentiation between the causal role of pre-existing systemic inflammation in UC onset and the consequential effect of UC progression on systemic inflammation. This distinction has important clinical implications when exploring novel therapeutic targets or identifying predictors of drug response or prognostic markers of future disease course for UC.

By employing genetic variants as instrumental variables for an exposure [eg, levels of a specific cytokine], Mendelian randomisation [MR] analysis can strengthen causal inference by minimising unobserved confounding and diminishing reverse causality.^10^ Because genetic variants are randomly allocated at conception, under certain assumptions the MR framework mimics randomised controlled trials and enables more robust causal inference than traditional epidemiological techniques. Here we conducted observational and MR analysis to provide different tiers of evidence to elaborate the exact role of proteomic signatures of systemic inflammation in the onset of UC.

## 2. Methods

### 2.1. Study design

**Figure 1.**
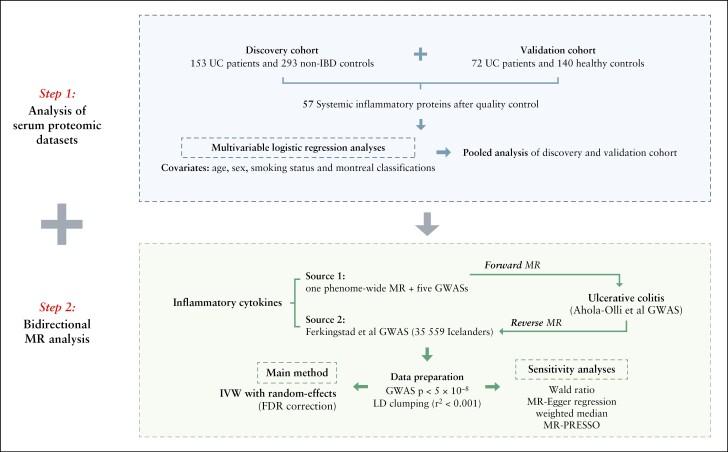
Schematic diagram of the study design. UC, ulcerative colitis; IBD, inflammatory bowel disease; MR, Mendelian randomisation; GWAS, genome-wide association study; FDR, false-discovery rate; IVW, inverse variance weighted method; LD, linkage disequilibrium.

In this study [Figure 1], we first synthesised serum proteomic profiling data from two published multicentre observational studies^11,12^ to examine the associations between systemic inflammatory proteins and the risk of UC. The case-control study was adopted as discovery dataset and the prospective case-cohort study as validation dataset. Next, bidirectional two-sample MR was performed to comprehensively examine the associations between circulating inflammatory proteins and UC risk from both forward and reverse directions.

### 2.2. Two-stage and pooled analyses of serum proteomic data from multicentred observational studies

#### 2.2.1. Study populations

The first discovery case-control study included 159 UC patients and 293 non-IBD controls who were recruited across six clinical centres in Europe.^11^ Blood samples for protein profiles were collected at baseline, ie, at the time of recruitment. The proteins measured in this study were involved in various UC-related mechanisms, including inflammation, immune regulation, metabolism, and cell-cell signalling. The second prospective nested case-cohort study for validation included 72 preclinical UC cases and 140 matched healthy controls who were recruited by the Northern Sweden Health and Disease Study in Sweden [NSHDSS].^12^ Participants who developed UC later in life were identified by linking the Northern Sweden Health and Disease Study register dataset with the International Classification of Diseases code register of Region of Västerbotten, Sweden. Pre-diagnostic plasma samples from UC patients, and from matched controls who remained free from IBD during follow-up, were measured for 92 proteins related to inflammation.

#### 2.2.2. Quality control

To minimise inter- and between-study variations, the pre-processed proteomic data were unified as arbitrary units, ie, normalised protein expression [NPX] on a log_2_ scale. A high NPX represents high protein concentration and a low NPX represents low protein concentration. The limit of detection [LOD] for each protein probe was defined as the mean plus three standard deviations [SDs] of the negative controls. The proteins for which >50% of samples were below the LOD were excluded from the analysis. After quality control and compiling the proteins together, a total of 57 common proteins related to systemic inflammation were eligible to be analysed in 231 UC patients and 433 controls.

#### 2.2.3. Statistical analysis

We used multivariable logistic regression models, with age, sex, smoking status, and Montreal classification as covariates, to investigate associations between individual proteins and risk of UC in discovery and validation study populations separately. A pooled analysis of individual data from the discovery and validation studies was further conducted to compute a pooled effect estimate with its 95% confidence interval [CI], with adjustment for age, sex, smoking status, Montreal classification, and study as covariates, for the associations between inflammation proteins and the risk of UC.

### 2.3. Causal inference—bidirectional two-sample MR analysis

#### 2.3.1. Data source for inflammatory proteins

MR analysis was conducted for the 14 inflammation proteins which were implicated by replication of association in both discovery and replication datasets. In the forward MR, single nucleotide polymorphisms [SNPs] strongly associated with systemic inflammation protein levels [*p* <5 × 10^-8^] [known as protein quantitative trait loci, pQTLs] were obtained from a published phenome-wide Mendelian randomisation of plasma proteosome^13^ and five genome-wide association studies [GWASs]^14–18^ [Supplementary Table 1]. In the reverse MR, genetic associations were derived from the most recent and largest GWAS of plasma proteins measured with 4907 aptamers of 35 559 Icelanders.^17^ Linkage disequilibrium [LD] was calculated based on the 1000 Genomes European reference panel, and genetic variants without LD [r^2^ ≤0.001 and clump window >10 000 kb] were finally selected as independent instrumental variables [IVs]. The selected genetic IVs were further classified as either 1] cis-pQTLs located in the vicinity of the encoding gene [defined as ≤500 kb from the leading pQTL of the test protein], or 2] trans-pQTLs located outside this window. MR analyses separately using cis- and trans-acting pQTLs and combined overall analyses were performed to test the robustness of the MR findings. Detailed information on the SNPs used as genetic IVs for inflammation proteins is presented in Supplementary Table 2.

#### 2.3.2. Data source for ulcerative colitis

Genetic associations between the cytokines-related SNPs and UC were obtained from the largest GWAS summary data of the international IBD genetic consortium [IIBDGC] study, which included 6968 UC cases and 21 770 population controls of European descent. Genetic variants associated with UC at the genome-wide significance level [*p* <5 × 10^-8^]. After pruning SNPs in linkage disequilibrium [r^2^ ≤0.001 and clump window >10 000 kb], 42 SNPs were selected as genetic IVs of UC to be used in reverse MR analyses [Supplementary Table 3].

#### 2.3.3. Two-sample MR analysis

For both the forward [the effect of circulating cytokines on UC] and reverse [the effect of genetic liability to UC on circulating cytokine levels] two-sample MR analyses, the inverse-variance weighted method with random effects was used as the main method. Four sensitivity analyses were conducted for supplement, including the weighted median,^10^ MR-Egger regression,^19^ MR-PRESSO,^20^ and leave-one-out analysis. Wald ratio was calculated for every single SNP to estimate the association between exposure and outcome. The weighted median method can provide consistent causal estimates if more than 50% of the weight comes from valid instrumental variables.^10^ MR-Egger can generate estimates after correcting for horizontal pleiotropy; however, this method compromises statistical power.^19^ MR-PRESSO can detect outlying instrumental variables and provide causal estimates after removal of these outliers.^20^ Cochrane’s Q value was used to assess the heterogeneity among estimates of genetic instruments, and the *p-*value for intercept in MR-Egger was used to detect horizontal pleiotropy.^19^ The strength of instruments was assessed by calculating F statistics [F <10 was deemed a weak instrument].^21^ The threshold of statistical significance was corrected by false-discovery rate [FDR] for multiple comparison: FDR <0.05 was regarded significant, and *p <*0.05 but not survived FDR was regarded suggestive significant. All statistical analyses were two-sided and performed in R 4.0.3 software using the R packages ‘TwoSampleMR’^22^ and ‘MR-PRESSO’.^20^

All studies were approved by the respective institutional review boards and conducted with appropriate ethical criteria in each country and in accordance with the Declaration of Helsinki.

## 3. Results

The basic characteristics of the study populations are presented in Table 1. In the discovery cohort, the mean age of participants was 37.3 vs 32.4 years, and the proportion of males was 57.9% vs 45.4% in UC patients and controls. In the validation cohort, the mean age was 48.3 vs 48.1 years, and the proportion of males was 47.2% vs 45.7% in UC patients and controls. The detailed results of individual and pooled analyses of serum proteomic profiling data for 57 systemic inflammatory protein markers from discovery and validation datasets are summarised in Supplementary Table 4.

**Table 1.** Characteristics of two observatory studies in secondary analysis of serum proteomic profiling data

Clinical characteristics	Discovery cohort			Validation cohort			Pooled		
	UC [*n* = 159]	Controls [*n* = 293]	*p-*value	UC [*n* = 72]	Controls [*n* = 140]	*p-*value	UC [*n* = 231]	Controls [*n* = 433]	*p-*value
Age, mean [SD]	37.3 [14.3]	32.4 [13.8]	<0.001	48.3 [10.8]	48.1 [10.7]	0.901	40.7 [14.2]	37.5 [14.9]	0.006
Sex, male, *n* [%]	92 [57.9]	133 [45.4]	0.015	34 [47.2]	64 [45.7]	0.950	126 [54.5]	197 [45.5]	0.032
Smoking status, *n* [%]									
Non-smoker	145 [91.2]	226 [77.1]	<0.001	50 [69.4]	105 [75.0]	0.241	195 [84.4]	331 [76.4]	0.003
Smoker	14 [8.8]	53 [18.1]		22 [30.6]	32 [22.9]		36 [15.6]	85 [19.6]	
Missing	0	14 [4.8]		0	3 [2.1]		0	17 [3.9]	
Disease extent for UC at diagnosis, *n* [%]									
Proctitis [E1]	39 [24.5]	-		16 [22.2]	-		55 [23.8]	-	
Left-sided colitis [E2]	47 [29.6]	-		28 [38.9]	-		75 [32.5]	-	
Extensive colitis [E3]	63 [39.6]	-		28 [38.9]	-		91 [39.4]	-	
Not available	10 [6.3]			0 [0]			10 [4.3]		

UC, ulcerative colitis; IBD, inflammatory bowel disease; SD, standard deviation.

**Figure 2.**
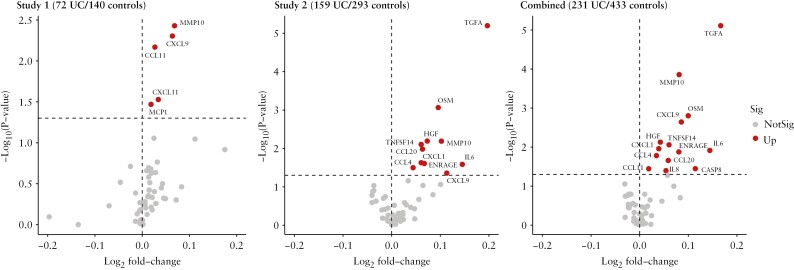
Adjusted results for analyses of serum proteomic data from discovery and validation datasets. UC, ulcerative colitis; MMP10, matrix metallopeptidase 10; CXCL, C-X-C motif chemokine ligand; CCL, C-C motif chemokine ligand; MCP1, ie, CCL2; TGFA, transforming growth factor alpha; OSM, oncostatin M; HGF, hepatocyte growth factor; TNFSF, TNF [tumour necrosis factor] superfamily member; IL, interleukin; ENRAGE, ie, S100A12, S100 calcium-binding protein A12; CASP8, caspase 8; Sig, significant.

In the discovery dataset, 11 systemic inflammatory proteins were found to be significantly higher in UC patients than controls after correction of multiple testing by FDR, including transforming growth factor alpha [TGFA] [log_2_fold change [log_2_FC] = 0.20, *p *= 1.16 × 10^-4^], oncostatin M [OSM] [log_2_FC = 0.10, *p *= 0.003], hepatocyte growth factor [HGF] [log_2_FC = 0.07, *p *= 0.015], matrix metallopeptidase 10 [MMP10] [log_2_FC = 0.10, *p *= 0.015], TNF superfamily member 14 [TNFSF14] [log_2_FC = 0.06, *p *= 0.016], IL6 [log_2_FC = 0.15, *p *= 0.021], C-X-C motif chemokine ligand [CXCL] 1 [log_2_FC = 0.06, *p *= .015], CCL20 [log_2_FC = 0.06, *p *= 0.024], extracellular newly identified RAGE-binding protein [EN-RAGE] [log_2_FC = 0.07, *p *= 0.025], CXCL9 [log_2_FC = 0.11, *p *= 0.030], and CCL4 [log2FC = 0.04, *p *= 0.032] [Figure 2]. In the validation dataset, two out of 11 protein markers were successfully replicated in a prospective study design: MMP10 [log_2_FC = 0.07, *p *= 0.015] and CXCL9 [log_2_FC = 0.06, *p *= 0.015], and three more cytokines, including CXCL11 [log_2_FC = 0.03, *p *= 0.035], CCL11 [log_2_FC = 0.03, *p *= 0.016], and monocyte chemoattractant protein-1 [MCP1] [log2FC = 0.02, *p *= 0.037], were additionally reported, indicating that individuals who developed UC during the follow-up period had significantly higher serum levels of these inflammatory markers at baseline [Figure 2].

In the pooled analyses of the two datasets, 14 out of 57 systemic inflammatory makers showed significant associations with UC risk after correction of multiple testing by FDR, including TGFA [log_2_FC = 0.17, *p *= 1.16 × 10^-4^], MMP10 [log_2_FC = 0.08, *p* = 0.001], OSM [log_2_FC = 0.10, *p *= 0.010], CXCL1 [log_2_FC = 0.04, *p *= 0.021], CXCL9 [log_2_FC = 0.09, *p *= 0.010], HGF [log_2_FC = 0.04, *p *= 0.016], TNFSF14 [log_2_FC = 0.06, *p *= 0.019], IL6 [log_2_FC = 0.14, *p *= 0.021], ENRAGE [log2FC = 0.081, *p *= 0.021], CCL4 [log_2_FC = 0.03, *p *= 0.024], CCL20 [log_2_FC = 0.06, *p *= 0.029], CASP8 [log_2_FC = 0.114, *p *= 0.037], CCL11 [log_2_FC = 0.02, *p *= 0.037], and IL8 [log_2_FC = 0.05, *p *= 0.040] [Figure 2]. The robustness of these associations was strengthened in sensitivity analyses as follows: 1] stratified analysis of smoking status separately in two cohorts and combined, with adjustments for age, sex, Montreal classifications, and study effect [Supplementary Figures S1–3]; 2] stratified analysis of Montreal classifications and study in the validation cohort, with adjustments for age, sex, and smoking status [Supplementary Figure S4].

**Figure 3.**
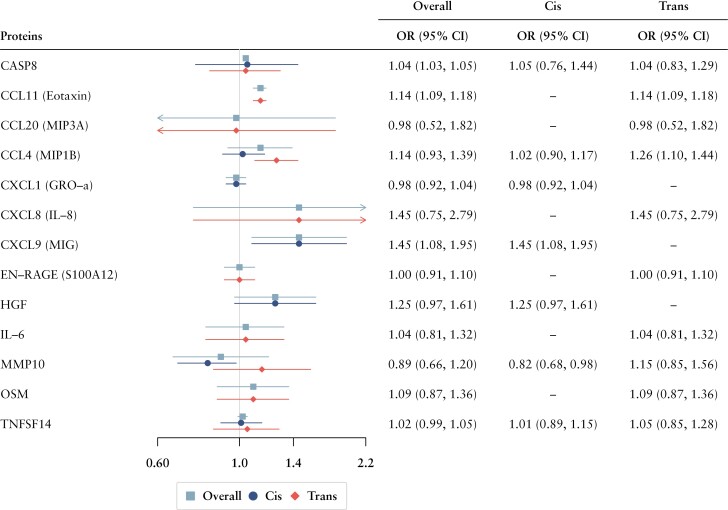
Associations of 13 cytokines with UC risk in forward MR analysis. MR, Mendelian randomisation; SNP, single nucleotide polymorphism; CI, confidence interval; CASP8, caspase 8; CCL, C-C motif chemokine ligand; MIP, major intrinsic protein of lens fibre; CXCL, C-X-C motif chemokine ligand; GRO, growth-regulated oncogene; IL, interleukin; MIG, monokine induced by gamma interferon; ENRAGE, ie, S100A12, S100 calcium-binding protein A12; HGF, hepatocyte growth factor; MMP10, matrix metallopeptidase 10; OSM, oncostatin M; TNFSF14, tumour necrosis factor superfamily member 14; OR, odds ratio; CI, confidence interval.

In both forward and reverse MR, the F statistics for used genetic instruments were all over 10, suggesting no substantial weak instrument bias [Supplementary Tables 2 and 3]. Detailed information and results of the forward MR analysis are shown in Figure 3 and summarised in Supplementary Table 5. Forward MR analyses were performed for 13 out of 14 inflammatory proteins identified from the observational study and for which there were available genetic IVs [we found no genetic eligible genetic IV for TGFA]. CXCL9 [OR 1.45, 95% CI 1.08, 1.95, *p *= 0.012; per SD increment] and CCL11 [OR 1.14, 95% CI 1.09, 1.18, *p* = 3.89 × 10^-10^; per SD increment] were suggested to have causal effect on the UC risk, respectively using only cis- or trans-acting pQTLs. CASP8 [OR 1.04, 95% CI 1.03, 1.05, *p *= 7.63 × 10^-19^; per SD increment] were additionally found to have significant associations with increased risk of UC, using both cis- and trans-acting pQTLs. In the reverse MR analysis, a total of 42 SNPs strongly associated with UC were included as genetic instruments [Supplementary Table 3]. Results from IVW MR analysis showed that genetic liability to UC was associated with none of the three inflammatory proteins [CXCL9, CCL11, CASP8] reported in forward MR. No indications of horizontal pleiotropy were detected by MR-Egger intercept test, and two outliers were found with CCL11 by the outlier test of MR-PRESSO analyses, but there were no significant differences before and after the correction. The results of reverse MR analysis are presented in Table 2.

**Table 2.** Associations between CXCL9, CCL11, and CASP8 levels and UC risk in the reverse MR analyses

Outcome	SNPs	Method	Beta coefficient [95% CI]	SE	*p*_effect_	*p*_heterogenicity_	*p*_intercept_
CXCL9	42	IVW	0.01 [-0.01, 0.02]	0.007	0.296	0.390	-
		MR Egger	0.02 [-0.02, 0.05]	0.016	0.347	0.361	0.587
		Weighted median	0.01 [-0.01, 0.03]	0.010	0.250	-	-
		MR-PRESSO	0.01 [-0.01, 0.02]	0.007	0.405	-	-
CCL11	42	IVW	0.01 [0, 0.03]	0.009	0.115	0.002	-
		MR Egger	0.02 [-0.02, 0.06]	0.021	0.398	0.001	0.860
		Weighted median	0.02 [-0.01, 0.04]	0.011	0.142	-	-
		MR-PRESSO	0.02 [0, 0.03]	0.008	0.002	-	-
CASP8	42	IVW	0.001 [-0.01, 0.01]	0.007	0.978	0.380	-
		MR Egger	0.01 [-0.02, 0.04]	0.016	0.529	0.359	0.490
		Weighted median	0.01 [-0.01, 0.03]	0.010	0.394	-	-
		MR-PRESSO	0 [-0.01, 0.01]	0.007	0.325	-	-

UC, ulcerative colitis; MR, Mendelian randomisation; SNPs, single nucleotide polymorphism; CI, confidence interval; SE, standard error; CXCL, C-X-C motif chemokine ligand; CCL, C-C motif chemokine ligand; CASP8, caspase 8; IVW, inverse variance weighted.

## 4. Discussion

The potential causal role of systemic inflammation in the pathogenesis of UC has not been fully established. A major advantage, laying the foundation of both novelty and rigour of this study, is the adoption of the MR approaches together with profiling of the serum proteome to provide different tiers of evidence to cross-validate the study findings. We compiled observational datasets and performed a pooled analysis of serum proteomic profiling data to derive primary clues for observational associations between inflammatory proteins and UC risk. The bidirectional MR study was then conducted based on inflammatory proteins first screened out, contributing to estimating both directions of association between inflammatory proteins and UC. These findings therefore provide us with major insights into a more precise causal inference about the exact role of cytokines in the onset or progress of UC. Five GWASs of systemic inflammatory proteins and one PheWAS-MR study of the plasma proteosome were employed as our data sources, comprehensively enlarging our investigation scope. Specifically, the pooled analysis of serum proteomic profiling data suggested that 14 systemic inflammatory proteins [TGFA, MMP10, OSM, CXCL1, CXCL9, HGF, TNFSF14, IL6, IL8, CCL4, CCL11, CCL20, CASP8, and ENRAGE] were significantly higher in preclinical or newly diagnosed UC patients than in controls. The forward MR analysis using cis-pQTLs and trans-pQTLs indicated that genetically predicted high circulating levels of CXCL9, CCL11, and CASP8 were associated with increased risk of UC; the reverse MR analysis did not find any influence of genetic predisposition to UC on the circulating levels of these inflammatory proteins.

Proteomic data employed in our analysis were derived from a case-control study [discovery cohort] and a prospective, nested case-cohort study [replication cohort], providing us with sufficient and enhanced strength of evidence. The discovery cohort recruited participants across six centres in Europe, to create and evaluate novel multi-protein panels based on known or suspected involvement in the pathogenesis of UC. The replication cohort was specially focused on UC developed later in life, which aimed to investigate the association between inflammatory proteins and future diagnosis of UC. This cohort of preclinical ulcerative colitis comprehensively measured the effects of 92 predefined inflammatory proteins,and was additionally employed to derive biosignature models and further validate the associations in a broader basis of the population. Given the inherent limitations of observational design and the fact that inflammation is well known to accompany diagnosis and often resolves quickly, these observed associations could only be implied as correlation rather than causation. We therefore took advantage of genetic instruments to proxy the long-term exposure to systemic inflammation and adopted an MR design to further explore any causal relationships.

High serum levels of CXCL9 were found to be causally associated with increased risk UC, in the pooled analysis of serum proteomic profiling data and forward MR analysis using pQTLs. CXCL9 is a chemokine of the C-X-C subfamily,^23^ expressed in macrophages under activation of interferon-gamma [IFN]-α^24^ and IFN-γ.^25^ Numerous studies have reported that CXCL9 levels are increased in several autoimmune diseases such as type 1 diabetes^26,27^ and systemic rheumatological disorders,^28–30^ indicating a potential association of CXCL9 with inflammatory diseases. A therapeutic strategy targeting the CXCL9 pathway has been indicated in a previous study,^31^ where curcumin phytochemicals were reported to inhibit the CXCL9 inflammatory cascade. Collectively, these findings underscore the importance of CXCL9 in disease onset and may pave a new way for the prevention and treatment of UC.

Serum CCL11 levels were also indicated to have a strong association with UC risk in the pooled analysis of proteomic data, and this observational association was further validated in MR analysis by using pQTLs. CCL11, also known as eotaxin-1, is another member of C-C motif chemokine subfamilies^32^ and can act as a chemoattractant of eosinophils and other leukocytes in inflammatory diseases.^33^ A previous case-control study, conducted in 35 patients with UC and 38 healthy controls, reported significantly higher serum levels of eotaxin [-1] in patients with UC, providing early observational evidence for the contribution of Eotaxin to the pathogenesis of UC.^34^ Another nested case-control study, including 137 UC cases and 38 healthy controls, found the levels of Eotaxin-1 to be elevated in patients with histologically active UC vs controls, using high-throughput technologies [Luminex-based multiplex testing] of 42 analytes, suggesting Eotaxin-1 to be an essential aetiological factor and a potential treatment target.^35^ Based on the effect of CCL11 mediated by activation of inflammatory cytokines and induction of tissue damage on the exacerbation of UC, some experimental studies have put an effort into investigating the CCL11-related path for treatment using antibody [anti-eotaxin-1] or constructing CCL11 deficient in sulphate sodium [DSS]-induced colitis mice,^36,37^ further demonstrating the potential value of the development of CCL11-targeted therapies.

Our study also reported that elevated levels of CASP8 were causally associated with increased risk of UC in both the pooled analysis of proteomic data and the forward MR analysis. CASP8 is a cysteine protease capable of inducing a process called ‘extrinsic apoptosis’ via death receptors [members of TNF subfamily],^38^ and it can also exert other non-apoptosis effects like regulating embryogenesis and cell proliferation.^39^ A recent cross-sectional study, including 40 patients with active UC and 21 healthy controls, suggested that CASP8 was a distinguishing protein in the systemic inflammatory protein profile, showing an increased level^40^ which possibly takes effect via interacting with inflammatory factors.^41^ Also, some studies provided supportive experimental evidence that the expression levels of CASP8 were significantly elevated in the colonic mucosa of rats with acetic acid [AA]-induced UC,^42^ which could be suppressed via the TLR4/NF-κB-related pathway [eg, betulin attenuates], TGF-β-related signalling [eg, cyclosporine],^43^ etc., revealing a ponderable perspective on the development of CASP8-targeted drugs or comprehensive treatments. Nonetheless, some studies indicated opposite findings that CASP8 was reduced in UC patients,^44^ and the deficiency of CASP8 could up-regulate the inflammasome activity via increasing the release of interacting protein 3 [RIP3].^45^ Given that experimental studies on animals are insufficient to achieve a broad extrapolation of conclusions, further studies of CASP8 in the human population are worth carrying out to confirm its pathological mechanisms in UC.

Numerous studies have proposed that lifestyle factors, such as dietary intakes, stress, and physical activities, have impacts on the onset and progress of UC, and have indicated that a healthy lifestyle is an effective strategy to reduce the systemic inflammation in UC.^46^ Diet with sufficient intake of fruits and vegetables may reduce the systemic inflammation,^47^ and long-term aerobic exercising was also reported to reduce serum levels of proinflammatory cytokines in older adults.^48^ Both of these findings suggested the role of healthy diet and physical activity in alleviating inflammatory diseases like UC. With establishing the role of these cytokines/chemokines in the progress of UC, efforts towards formulating pharmacological [potential drugs or phytochemicals] or non-pharmacological strategies [anti-inflammatory diet, exercise, or stress reduction] via the regulation of CXCL9, CCL11, and CASP8, should be considered as possible strategies for primary prevention of UC.

### 4.1. Limitations

Notwithstanding the strengths of our study, there are also some potential limitations. In this study, we only conducted MR analysis with 13 inflammatory proteins that had been identified by a pooled analysis of two serum proteomic datasets, which constrained the range of cytokines assessed such that we were not able to verify potentially effective cytokines reported in our analyses of observational studies [eg, TGFA] or suggested by previous studies [eg, IL23, IL7, etc.]. Another limitation of our study is that using SNPs as instruments to deduce causality in MR analyses may risk horizontal pleiotropy, particularly for protein markers proxied by a few SNPs. In addition, the confinement of population to European descent is important in considering the generaliability of our findings to other populations, due to racial differences.

## 4.2. Conclusions

Overall, our study provides a hierarchy of evidence for the causal involvement of dysregulation of CXCL9, CCL11, and CASP8 in the pathogenesis of UC, from both observational and genetic analyses. Our study reveals that long-term pre-existing high levels of CXCL9, CXCL11, and CASP8 may increase the risk of UC without reverse causal interference. Our findings support the primary importance of dysregulation cytokines in the pathogenesis of UC, and provide novel perspectives for treatment strategies and drug development based on mechanisms related to those cytokines and chemokines.

The analysis results of this study are included in this published article and its Supplementary information files. The UK Biobank dataset can be accessed through their access application process.

## Supplementary Material

jjac191_suppl_Supplementary_Material
