# Supplementary material for: Bidirectional Mendelian Randomisation Analysis Provides Evidence for the Causal Involvement of Dysregulation of CXCL9, CCL11 and CASP8 in the Pathogenesis of Ulcerative Colitis
Source: J Crohns Colitis. 2022 Dec 28;17(5):777–85. doi: 10.1093/ecco-jcc/jjac191 (PMC10155748; doi:10.1093/ecco-jcc/jjac191)
Supplement: jjac191_suppl_Supplementary_Material [file jjac191_suppl_supplementary_material.docx]

**Supplementary Materials of Tables and Figures**

**Supplementary Table 1. Included studies and consortium in Mendelian randomization analyses.**

Abbreviations: IV, instrumental variables; GWAS, genome-wide association study; OSM, oncostatin M; HGF, hepatocyte growth factor; CXCL, C-X-C motif chemokine ligand; GRO, growth-regulated oncogene; IL, interleukin; MIG, monokine induced by gamma interferon; CCL, C-C motif chemokine ligand; MIP, major intrinsic protein of lens fiber; TNFSF14, TNF superfamily member 14; MMP10, matrix metallopeptidase 10; CASP8, caspase 8; ENRAGE,i.e. S100A12, S100 calcium binding protein A12; UC, ulcerative colitis.

**Supplementary Table 2. Details of genetic instruments for inflammatory cytokines (with genome-wide significant SNPs).**

Abbreviations: SNP, single nucleotide polymorphism; Chr, chromosome; Pos, position; EA, effect allele; NEA, non-effect allele; EAF, effect allele frequency; SE, standard error; CXCL, C-X-C motif chemokine ligand; CCL, C-C motif chemokine ligand; MIP, major intrinsic protein of lens fiber; GRO, growth-regulated oncogene; IL, interleukin; MIG, monokine induced by gamma interferon; HGF, hepatocyte growth factor; MMP10, matrix metallopeptidase 10; OSM, oncostatin M; CASP8, caspase 8; ENRAGE,i.e. S100A12, S100 calcium binding protein A12; TNFSF14, TNF superfamily member 14.

**Supplementary Table 3. Details of genetic instruments for ulcerative colitis (at genome-wide significant threshold).**

Abbreviations: SNP, single nucleotide polymorphism; Chr, chromosome; EAF, effect allele frequency; SE, standard error.

**Supplementary Table 4.** **Results of separate and pooled analyses of serum proteomic profiling data for 57 systemic inflammation proteins from discovery and validation datasets.**

Abbreviations: SE, standard error; FC, fold change; ADA, adenosine deaminase; CASP8, caspase 8; CCL, C-C motif chemokine ligand; CDCP1, CUB domain containing protein 1; CSF, colony stimulating factor; CST, cystatin; CX3CL, C-X3-C motif chemokine ligand; CXCL, C-X-C motif chemokine ligand; DNER, delta/notch like EGF repeat containing; ENRAGE, i.e. S100A12, S100 calcium binding protein A12; FGF, fibroblast growth factor; Flt3L, fms related receptor tyrosine kinase 3 ligand; HGF, hepatocyte growth factor; IL, interleukin; IL10RB, interleukin 10 receptor subunit beta; TGF, transforming growth factor; MCP1,monocyte chemotactic protein, i.e., CCL2; MIP, major intrinsic protein of lens fiber; MMP, matrix metallopeptidase; OPG, i.e. TNFRSF11B, TNF receptor superfamily member 11b; OSM, oncostatin M; SCF, stem cell factor; SIRT2, sirtuin 2; STAMPB, stimulatory transmembrane protein; TGFA, transforming growth factor alpha; TNF, tumor necrosis factor; TNFRSF, TNF receptor superfamily member; TRAIL, i.e. TNFSF10, TNF superfamily member 10; TRANCE, i.e. TNFSF11; TWEAK, i.e. TNFSF12; uPA, urokinase; VEGFA, vascular endothelial growth factor A.

**Supplementary Table 5.** Results of the associations between inflammatory cytokines and ulcerative colitis using forward Mendelian randomization analyses.

Notes: Inverse weighted method was adopted for cytokines with more than 2 SNPs, and Wald ratio method for cytokines with only 1 SNP.

Abbreviations: nSNP, number of single nucleotide polymorphism; OR, odds ratio; CI, confidence interval; FDR, false discovery rate; CCL, C-C motif chemokine ligand; MIP, major intrinsic protein of lens fiber; CXCL, C-X-C motif chemokine ligand; GRO, growth-regulated oncogene; IL, interleukin; MIG, monokine induced by gamma interferon; HGF, hepatocyte growth factor; MMP10, matrix metallopeptidase 10; OSM, oncostatin M; CASP8, caspase 8; ENRAGE,i.e. S100A12, S100 calcium binding protein A12; TNFSF14, TNF superfamily member 14.

**Figure S1.** Stratified analysis of smoking status in the discovery cohort with adjustments of age, sex and Montreal classifications and study effect.

Abbreviations: UC, ulcerative colitis; TGFA, transforming growth factor alpha; OSM, oncostatin M; MMP10, matrix metallopeptidase 10; CCL, C-C motif chemokine ligand; HGF, hepatocyte growth factor; IL, interleukin; CXCL, C-X-C motif chemokine ligand; TNFSF, TNF superfamily member; VEGFA, vascular endothelial growth factor A; ENRAGE, i.e. S100A12, S100 calcium binding protein A12; LAP.TGFbeta, transforming growth factor beta; sig, significant.

**Figure S2.** Stratified analysis of smoking status in the validation cohort with adjustments of age, sex and Montreal classifications and study effect.

Abbreviations: UC, ulcerative colitis; CXCL, C-X-C motif chemokine ligand; CCL, C-C motif chemokine ligand; MMP10, matrix metallopeptidase 10; CASP8, caspase 8; SCF, stem cell factor; sig, significant.

**Figure S3.** Stratified analysis of smoking status in both cohorts with adjustments of age, sex and Montreal classifications and study effect.

Abbreviations: UC, ulcerative colitis; TGFA, transforming growth factor alpha; OSM, oncostatin M; MMP10, matrix metallopeptidase 10; CXCL, C-X-C motif chemokine ligand; CCL, C-C motif chemokine ligand; ENRAGE, i.e. S100A12, S100 calcium binding protein A12; HGF, hepatocyte growth factor; IL, interleukin; TNFSF, TNF superfamily member; VEGFA, vascular endothelial growth factor A; TGF, transforming growth factor; MCP1, monocyte chemotactic protein, i.e., CCL2; LAP.TGFbeta, transforming growth factor beta; sig, significant.

**Figure S4.** Stratified analysis of Montreal classifications and study in the validation cohort with adjustments of age, sex and smoking effect.

Abbreviations: UC, ulcerative colitis; CXCL, C-X-C motif chemokine ligand; STAMPB, stimulatory transmembrane protein; MMP10, matrix metallopeptidase 10; CCL, C-C motif chemokine ligand; sig, significant.

**Supplementary Table 1. Included studies and consortium in Mendelian randomization analyses.**

| **Exposure/Outcome** | **Numbers of IV** | **Source** | **Participants** | **Adjustment** |
| --- | --- | --- | --- | --- |
| OSM | 1 | Sun et al GWAS^1^ | 3,301 healthy participants from the INTERVAL study of European ancestry | Age, sex, duration between blood draw and processing (binary, ≤1 day/>1day) and the first three principal components |
| HGF | 1 |  |  |  |
| CXCL1 (GRO-a) | 1 |  |  |  |
| CXCL11 (Eotaxin) | 2 |  |  |  |
| IL-6 | 2 | Folkersen et al GWAS^2^ | 30,931 participants across 14 studies of European ancestry | Age, sex, population structure, and study-specific parameters |
| CXCL8 (IL-8) | 2 |  |  |  |
| CXCL9 (MIG) | 1 | Ahola-Olli AV et al GWAS^3^ | up to 8,293 individuals of European ancestry | Age, sex, body mass index, and the first ten genetic principal components |
| CCL4 (MIP1B) | 2 | Suhre et al GWAS^1^, Folkersen et al GWAS^2^ | Suhre et al GWAS: 1000 European participants from the KORA F4 study cohort and 384 European participants of cross-sectional case-control study (QMDiab)  Folkersen et al GWAS: 30,931 participants across 14 studies of European ancestry | Suhre et al GWAS: Age, sex, and body mass index  Folkersen et al GWAS: Age, sex, population structure, and study-specific parameters |
| CCL20 (MIP3A) | 1 | Ferkingstad et al GWAS^4^ | 35,559 Icelanders from two main projects: the Icelandic Cancer Project and various genetic programs at deCODE genetics | Age and sex |
| TNFSF14 | 2 | Sun et al GWAS^1^, Folkersen et al GWAS^2^ | Sun et al GWAS: 3,301 healthy participants from the INTERVAL study of European ancestry  Folkersen et al GWAS: 30,931 participants across 14 studies of European ancestry | Sun et al GWAS: Age, sex, duration between blood draw and processing (binary, ≤1 day/>1day) and the first three principal components  Folkersen et al GWAS: Age, sex, population structure, and study-specific parameters |
| MMP10 | 2 |  |  |  |
| CASP8 | 2 | Folkersen et al GWAS^2^ | 30,931 participants across 14 studies of European ancestry | Age, sex, population structure, and study-specific parameters |
| ENRAGE (S100A12) | 1 | Sun et al GWAS^1^ | 3,301 healthy participants from the INTERVAL study of European ancestry | Age, sex, duration between blood draw and processing (binary, ≤1 day/>1day) and the first three principal components |
| UC | 42 | Liu et al GWAS^5^ | 6,968 UC cases and 21,770 controls of European populations | Population structure and study-specific parameters |

**Supplementary Table 2. Details of genetic instruments for inflammatory cytokines (with genome-wide significant SNPs).**

| **Protein** | **SNP** | **Cis/Trans** | **Chr** | **Pos** | **EA** | **NEA** | **EAF** | **Sample size** | **R-square** | **F-statistics** | **Wald Ratio** | | |
| --- | --- | --- | --- | --- | --- | --- | --- | --- | --- | --- | --- | --- | --- |
|  |  |  |  |  |  |  |  |  |  |  | **Beta** | **SE** | **P value** |
| CXCL11 (Eotaxin) | rs12075 | trans | 1 | 159175354 | A | G | 0.584 | 3301 | 0.025 | 86.26 | 0.229 | 0.025 | 8.50E-20 |
|  | rs2228467 | trans | 3 | 42906116 | C | T | 0.067 | 3301 | 0.039 | 132.13 | 0.555 | 0.049 | 4.50E-30 |
| CCL20 (MIP3A) | rs6542680 | trans | 2 | 3592552 | T | C | 0.816 | 35559 | 0.003 | 95.25 | -0.094 | 0.010 | 1.61E-20 |
| CCL4 (MIP1B) | rs6808835 | trans | 3 | 46449864 | T | G | 0.138 | 1384 | 0.044 | 63.82 | 0.430 | 0.064 | 3.49E-11 |
|  | rs6607368 | cis | 17 | 34819136 | A | C | 0.800 | 30931 | 0.083 | 2808.00 | 0.510 | 0.044 | 6.30E-31 |
| CXCL1 (GRO-a) | rs2115691 | cis | 4 | 74743691 | G | C | 0.240 | 3301 | 0.203 | 840.86 | 0.746 | 0.026 | 2.00E-178 |
| CXCL8 (IL-8) | rs7655660 | trans | 4 | 74589597 | A | G | 0.068 | 30931 | 0.004 | 139.16 | -0.188 | 0.024 | 2.67E-15 |
|  | rs972492 | trans | 5 | 37903927 | C | A | 0.857 | 30931 | 0.003 | 93.13 | 0.111 | 0.020 | 3.83E-08 |
| CXCL9 (MIG) | rs7655660 | trans | 4 | 74589597 | T | C | 0.247 | 8293 | 0.010 | 85.14 | -0.165 | 0.026 | 1.13E-10 |
| HGF | rs5745695 | cis | 7 | 81358075 | A | G | 0.760 | 3301 | 0.014 | 45.97 | 0.194 | 0.029 | 1.20E-11 |
| IL-6 | rs2228145 | trans | 1 | 154426970 | C | A | 0.379 | 30931 | 0.014 | 450.63 | 0.175 | 0.012 | 3.34E-45 |
|  | rs4959106 | trans | 6 | 32583159 | C | T | 0.462 | 30931 | 0.003 | 104.48 | 0.082 | 0.014 | 2.37E-09 |
| MMP10 | rs17860955 | cis | 11 | 102649482 | C | T | 0.022 | 3301 | 0.032 | 108.38 | -0.869 | 0.087 | 1.40E-23 |
|  | rs492602 | trans | 19 | 49206417 | G | A | 0.450 | 30931 | 0.010 | 303.01 | 0.140 | 0.024 | 7.80E-09 |
| OSM | rs74480769 | trans | 5 | 40972211 | G | A | 0.032 | 3301 | 0.017 | 57.47 | -0.528 | 0.073 | 5.40E-13 |
| CASP8 | rs35550815 | cis | 2 | 202150914 | G | A | 0.128 | 30931 | 0.008 | 260.17 | 0.194 | 0.018 | 5.96E-26 |
|  | rs10424405 | trans | 19 | 54321933 | G | A | 0.220 | 30931 | 0.029 | 916.56 | 0.289 | 0.015 | 1.43E-79 |
| ENRAGE | rs62143206 | trans | 19 | 54326212 | T | G | 0.213 | 3301 | 0.158 | 616.97 | 0.685 | 0.028 | 4.30E-136 |
| TNFSF14 | rs344560 | cis | 19 | 6665020 | C | T | 0.960 | 30931 | 0.043 | 1396.46 | 0.750 | 0.086 | 3.00E-18 |
|  | rs62143198 | trans | 19 | 54320939 | A | G | 0.215 | 3301 | 0.032 | 107.50 | 0.306 | 0.030 | 5.10E-24 |

**Supplementary Table 3. Details of genetic instruments for ulcerative colitis (at genome-wide significant threshold).**

| **SNP** | **Sample** | **Chr** | **Position** | **Effect Allele** | **Other Allele** | **EAF** | **Beta** | **SE** | **P value** | **R-square** | **F-statistic** |
| --- | --- | --- | --- | --- | --- | --- | --- | --- | --- | --- | --- |
| rs1886731 | 27432 | 1 | 2472081 | C | T | 0.48 | -0.141 | 0.022 | 2.25E-10 | 0.010 | 274.96 |
| rs34920465 | 27432 | 1 | 22700351 | G | A | 0.17 | -0.193 | 0.029 | 2.93E-11 | 0.011 | 291.40 |
| rs10737481 | 27432 | 1 | 20171514 | G | T | 0.56 | 0.250 | 0.022 | 4.37E-31 | 0.031 | 871.69 |
| rs10917547 | 27432 | 1 | 20143142 | T | A | 0.36 | -0.173 | 0.022 | 5.51E-15 | 0.014 | 383.59 |
| rs7523335 | 27432 | 1 | 8180210 | A | G | 0.18 | -0.170 | 0.029 | 2.29E-09 | 0.009 | 236.03 |
| rs1317209 | 27432 | 1 | 20140036 | A | G | 0.19 | 0.146 | 0.027 | 3.47E-08 | 0.007 | 181.16 |
| rs11209026 | 27432 | 1 | 67705958 | A | G | 0.06 | -0.562 | 0.052 | 1.58E-27 | 0.036 | 1013.36 |
| rs35730213 | 27432 | 1 | 200874229 | C | G | 0.27 | -0.167 | 0.025 | 8.81E-12 | 0.011 | 304.91 |
| rs3024493 | 27432 | 1 | 206943968 | A | C | 0.17 | 0.236 | 0.028 | 1.09E-17 | 0.016 | 438.01 |
| rs12612675 | 27432 | 2 | 219133137 | G | A | 0.40 | 0.123 | 0.022 | 1.98E-08 | 0.007 | 200.65 |
| rs4676410 | 27432 | 2 | 241563739 | A | G | 0.20 | 0.208 | 0.028 | 2.46E-13 | 0.014 | 385.09 |
| rs10182512 | 27432 | 2 | 61189469 | A | G | 0.35 | 0.161 | 0.022 | 5.19E-13 | 0.012 | 327.37 |
| rs9823546 | 27432 | 3 | 49705512 | A | T | 0.31 | 0.177 | 0.022 | 2.29E-15 | 0.013 | 372.63 |
| rs254559 | 27432 | 5 | 134444982 | A | C | 0.40 | 0.124 | 0.022 | 7.63E-09 | 0.007 | 203.95 |
| rs56167332 | 27432 | 5 | 158827769 | A | C | 0.34 | 0.152 | 0.023 | 5.30E-11 | 0.010 | 287.40 |
| rs144582178 | 27432 | 6 | 32481800 | C | T | 0.67 | -0.276 | 0.028 | 4.06E-23 | 0.034 | 956.19 |
| rs117292830 | 27432 | 6 | 31218268 | A | G | 0.03 | 0.615 | 0.070 | 2.20E-18 | 0.022 | 617.40 |
| rs45627734 | 27432 | 6 | 31474884 | A | G | 0.03 | 0.406 | 0.063 | 1.47E-10 | 0.010 | 265.70 |
| rs144515162 | 27432 | 6 | 32455083 | G | A | 0.67 | -0.232 | 0.027 | 2.69E-17 | 0.024 | 668.78 |
| rs183231933 | 27432 | 6 | 32524320 | T | C | 0.24 | -0.371 | 0.031 | 1.71E-32 | 0.050 | 1450.11 |
| rs148844907 | 27432 | 6 | 31628397 | A | T | 0.01 | 1.341 | 0.109 | 7.17E-35 | 0.036 | 1012.73 |
| rs7752873 | 27432 | 6 | 106579332 | T | C | 0.14 | 0.182 | 0.030 | 1.83E-09 | 0.008 | 220.55 |
| rs9272514 | 27432 | 6 | 32606385 | T | C | 0.30 | -0.402 | 0.027 | 4.00E-51 | 0.068 | 1997.34 |
| rs28383456 | 27432 | 6 | 32609453 | T | C | 0.33 | -0.337 | 0.026 | 1.07E-39 | 0.050 | 1450.38 |
| rs115312361 | 27432 | 6 | 32488215 | C | A | 0.33 | -0.324 | 0.029 | 9.44E-29 | 0.046 | 1335.30 |
| rs6933404 | 27432 | 6 | 137959235 | C | T | 0.22 | 0.167 | 0.025 | 3.68E-11 | 0.010 | 265.08 |
| rs10272963 | 27432 | 7 | 107486902 | T | C | 0.43 | -0.172 | 0.022 | 1.69E-15 | 0.015 | 403.65 |
| rs798502 | 27432 | 7 | 2789880 | C | A | 0.28 | -0.136 | 0.024 | 1.21E-08 | 0.007 | 206.10 |
| rs2301989 | 27432 | 7 | 107443871 | A | G | 0.40 | -0.141 | 0.022 | 8.55E-11 | 0.010 | 264.28 |
| rs3829111 | 27432 | 9 | 139269483 | A | G | 0.42 | 0.156 | 0.021 | 2.89E-13 | 0.012 | 329.13 |
| rs1887428 | 27432 | 9 | 4984530 | C | G | 0.62 | -0.177 | 0.022 | 3.36E-15 | 0.015 | 411.00 |
| rs4574921 | 27432 | 9 | 117538334 | T | C | 0.74 | 0.151 | 0.026 | 4.24E-09 | 0.009 | 242.80 |
| rs7911680 | 27432 | 10 | 101293468 | C | A | 0.49 | -0.172 | 0.021 | 8.27E-16 | 0.015 | 411.67 |
| rs484356 | 27432 | 11 | 114406639 | G | C | 0.33 | -0.134 | 0.023 | 3.95E-09 | 0.008 | 219.54 |
| rs2212434 | 27432 | 11 | 76281593 | T | C | 0.46 | 0.142 | 0.021 | 2.46E-11 | 0.010 | 277.56 |
| rs12817473 | 27432 | 12 | 68497408 | G | A | 0.38 | 0.191 | 0.022 | 1.71E-18 | 0.017 | 479.76 |
| rs1359946 | 27432 | 13 | 27536972 | A | G | 0.20 | 0.158 | 0.027 | 3.84E-09 | 0.008 | 220.89 |
| rs9891174 | 27432 | 17 | 38031802 | A | T | 0.47 | 0.145 | 0.021 | 7.17E-12 | 0.010 | 290.36 |
| rs6062496 | 27432 | 20 | 62329099 | A | G | 0.57 | 0.158 | 0.022 | 1.47E-12 | 0.012 | 339.83 |
| rs6017342 | 27432 | 20 | 43065028 | C | A | 0.54 | 0.191 | 0.024 | 1.38E-15 | 0.018 | 506.31 |
| rs7282490 | 27432 | 21 | 45615741 | A | G | 0.60 | -0.140 | 0.021 | 7.08E-11 | 0.009 | 260.51 |
| rs137845 | 27432 | 22 | 50439430 | G | A | 0.51 | 0.118 | 0.021 | 2.38E-08 | 0.007 | 192.23 |

**Supplementary Table 4. Results of separate and pooled analyses of serum proteomic profiling data for 57 systemic inflammation proteins from discovery and validation datasets.**

| **Cytokines** | **Combined** | | | |  | **Discovery cohort** | | | |  | **Validation cohort** | | | |
| --- | --- | --- | --- | --- | --- | --- | --- | --- | --- | --- | --- | --- | --- | --- |
|  | **Beta** | **SE** | **log2FC** | **P-value** |  | **Beta** | **SE** | **log2FC** | **P-value** |  | **Beta** | **SE** | **log2FC** | **P-value** |
| ADA | 0.016 | 0.141 | 0.01 | 0.910 |  | 0.071 | 0.158 | 0.02 | 0.653 |  | -0.116 | 0.347 | -0.01 | 0.739 |
| CASP8 | 0.137 | 0.065 | 0.11 | 0.036 |  | 0.112 | 0.072 | 0.08 | 0.119 |  | 0.275 | 0.178 | 0.17 | 0.121 |
| CCL11 | 0.298 | 0.142 | 0.02 | 0.036 |  | 0.250 | 0.156 | 0.03 | 0.108 |  | 1.319 | 0.487 | 0.03 | 0.007 |
| CCL19 | -0.064 | 0.089 | -0.01 | 0.471 |  | -0.108 | 0.106 | -0.01 | 0.308 |  | 0.024 | 0.170 | 0.00 | 0.887 |
| CCL20 | 0.183 | 0.080 | 0.06 | 0.022 |  | 0.244 | 0.103 | 0.06 | 0.017 |  | 0.136 | 0.135 | 0.04 | 0.316 |
| CCL23 | 0.112 | 0.117 | 0.01 | 0.342 |  | 0.158 | 0.128 | 0.03 | 0.218 |  | -0.129 | 0.304 | -0.01 | 0.671 |
| CCL25 | -0.125 | 0.133 | -0.02 | 0.350 |  | -0.257 | 0.178 | -0.04 | 0.148 |  | 0.110 | 0.216 | 0.02 | 0.611 |
| CCL4 | 0.281 | 0.117 | 0.03 | 0.017 |  | 0.314 | 0.142 | 0.04 | 0.027 |  | 0.307 | 0.224 | 0.03 | 0.172 |
| CD244 | -0.315 | 0.185 | -0.03 | 0.088 |  | -0.331 | 0.209 | -0.04 | 0.115 |  | -0.020 | 0.415 | 0.00 | 0.962 |
| CD40 | -0.164 | 0.151 | -0.01 | 0.278 |  | -0.208 | 0.169 | -0.01 | 0.219 |  | 0.238 | 0.373 | 0.01 | 0.524 |
| CD5 | 0.116 | 0.169 | 0.01 | 0.490 |  | -0.004 | 0.185 | 0.00 | 0.982 |  | 0.806 | 0.472 | 0.02 | 0.088 |
| CD6 | -0.141 | 0.136 | -0.02 | 0.299 |  | -0.210 | 0.155 | -0.04 | 0.174 |  | 0.276 | 0.325 | 0.03 | 0.395 |
| CDCP1 | -0.073 | 0.129 | 0.02 | 0.570 |  | -0.112 | 0.152 | 0.01 | 0.462 |  | 0.205 | 0.291 | 0.05 | 0.481 |
| CSF1 | 0.089 | 0.131 | 0.01 | 0.494 |  | 0.067 | 0.137 | 0.02 | 0.624 |  | 0.715 | 0.582 | 0.01 | 0.219 |
| CST5 | 0.023 | 0.165 | 0.00 | 0.892 |  | 0.057 | 0.199 | 0.01 | 0.773 |  | 0.251 | 0.335 | 0.01 | 0.455 |
| CX3CL1 | -0.046 | 0.154 | -0.01 | 0.765 |  | -0.076 | 0.179 | -0.01 | 0.673 |  | 0.301 | 0.343 | 0.01 | 0.380 |
| CXCL1 | 0.223 | 0.088 | 0.04 | 0.011 |  | 0.236 | 0.097 | 0.06 | 0.015 |  | 0.200 | 0.227 | 0.01 | 0.377 |
| CXCL10 | 0.054 | 0.091 | 0.01 | 0.553 |  | 0.039 | 0.111 | 0.02 | 0.723 |  | 0.189 | 0.168 | 0.01 | 0.262 |
| CXCL11 | 0.122 | 0.087 | 0.01 | 0.161 |  | 0.076 | 0.101 | 0.02 | 0.454 |  | 0.461 | 0.212 | 0.03 | 0.030 |
| CXCL5 | -0.041 | 0.091 | -0.01 | 0.653 |  | 0.035 | 0.113 | 0.00 | 0.755 |  | -0.208 | 0.160 | -0.02 | 0.194 |
| CXCL6 | -0.096 | 0.105 | -0.02 | 0.362 |  | -0.063 | 0.116 | -0.01 | 0.587 |  | -0.207 | 0.251 | -0.01 | 0.411 |
| CXCL9 | 0.232 | 0.076 | 0.09 | 0.002 |  | 0.188 | 0.083 | 0.11 | 0.024 |  | 0.607 | 0.216 | 0.06 | 0.005 |
| DNER | -0.227 | 0.162 | -0.02 | 0.160 |  | -0.167 | 0.175 | -0.02 | 0.343 |  | -0.286 | 0.464 | 0.00 | 0.538 |
| ENRAGE | 0.216 | 0.087 | 0.08 | 0.013 |  | 0.247 | 0.104 | 0.07 | 0.018 |  | 0.116 | 0.172 | 0.07 | 0.500 |
| FGF19 | -0.012 | 0.091 | -0.01 | 0.891 |  | -0.093 | 0.113 | -0.02 | 0.409 |  | 0.204 | 0.170 | 0.03 | 0.231 |
| FGF21 | 0.003 | 0.052 | 0.02 | 0.952 |  | 0.027 | 0.059 | 0.05 | 0.647 |  | -0.119 | 0.116 | -0.05 | 0.304 |
| Flt3L | -0.187 | 0.133 | -0.02 | 0.161 |  | -0.188 | 0.147 | -0.02 | 0.201 |  | -0.062 | 0.372 | 0.00 | 0.868 |
| HGF | 0.322 | 0.120 | 0.04 | 0.007 |  | 0.366 | 0.128 | 0.07 | 0.004 |  | -0.004 | 0.437 | 0.00 | 0.992 |
| IL10 | 0.130 | 0.101 | 0.08 | 0.198 |  | 0.172 | 0.110 | 0.10 | 0.117 |  | -0.077 | 0.306 | - | 0.801 |
| IL10RB | 0.110 | 0.168 | 0.00 | 0.514 |  | 0.081 | 0.185 | 0.01 | 0.662 |  | 0.583 | 0.457 | 0.01 | 0.202 |
| IL12B | -0.118 | 0.115 | -0.01 | 0.304 |  | -0.104 | 0.132 | -0.01 | 0.430 |  | 0.029 | 0.259 | -0.01 | 0.911 |
| IL18 | 0.039 | 0.126 | 0.01 | 0.754 |  | 0.100 | 0.146 | 0.02 | 0.494 |  | -0.042 | 0.267 | 0.00 | 0.875 |
| IL18R1 | 0.041 | 0.134 | 0.00 | 0.762 |  | 0.005 | 0.154 | 0.01 | 0.975 |  | 0.368 | 0.310 | 0.02 | 0.235 |
| IL6 | 0.173 | 0.069 | 0.14 | 0.012 |  | 0.174 | 0.069 | 0.15 | 0.012 |  | -535.986 | 886152.415 | - | 1.000 |
| IL7 | -0.010 | 0.129 | 0.01 | 0.940 |  | 0.046 | 0.163 | 0.00 | 0.777 |  | -0.123 | 0.227 | -0.07 | 0.589 |
| IL8 | 0.164 | 0.080 | 0.05 | 0.040 |  | 0.173 | 0.094 | 0.06 | 0.065 |  | 0.144 | 0.156 | 0.04 | 0.356 |
| TGF beta | 0.091 | 0.093 | 0.02 | 0.325 |  | 0.074 | 0.098 | 0.02 | 0.448 |  | 0.490 | 0.390 | 0.02 | 0.209 |
| MCP1 | 0.085 | 0.150 | 0.00 | 0.572 |  | -0.019 | 0.165 | 0.00 | 0.910 |  | 0.896 | 0.422 | 0.02 | 0.034 |
| MCP2 | 0.023 | 0.127 | 0.00 | 0.853 |  | 0.025 | 0.158 | 0.00 | 0.876 |  | 0.239 | 0.234 | 0.02 | 0.307 |
| MCP4 | 0.015 | 0.124 | 0.01 | 0.905 |  | 0.060 | 0.162 | 0.01 | 0.713 |  | 0.155 | 0.212 | 0.02 | 0.466 |
| MIP1 alpha | 0.132 | 0.113 | 0.05 | 0.243 |  | 0.184 | 0.151 | 0.05 | 0.224 |  | 0.128 | 0.179 | 0.05 | 0.475 |
| MMP1 | 0.021 | 0.080 | -0.01 | 0.792 |  | -0.023 | 0.093 | 0.00 | 0.802 |  | 0.125 | 0.166 | 0.02 | 0.452 |
| MMP10 | 0.373 | 0.098 | 0.08 | 0.000 |  | 0.301 | 0.108 | 0.10 | 0.005 |  | 0.717 | 0.247 | 0.07 | 0.004 |
| OPG | 0.024 | 0.148 | 0.00 | 0.871 |  | 0.066 | 0.161 | 0.01 | 0.680 |  | -0.199 | 0.444 | 0.00 | 0.655 |
| OSM | 0.239 | 0.076 | 0.10 | 0.002 |  | 0.332 | 0.093 | 0.10 | 0.000 |  | -0.016 | 0.144 | 0.00 | 0.912 |
| SCF | -0.150 | 0.112 | -0.03 | 0.181 |  | -0.142 | 0.123 | -0.03 | 0.248 |  | -0.013 | 0.292 | 0.00 | 0.964 |
| SIRT2 | 0.138 | 0.084 | 0.07 | 0.101 |  | 0.126 | 0.100 | 0.06 | 0.209 |  | 0.153 | 0.163 | 0.08 | 0.346 |
| STAMPB | 0.216 | 0.112 | 0.06 | 0.053 |  | 0.147 | 0.139 | 0.03 | 0.289 |  | 0.332 | 0.196 | 0.11 | 0.090 |
| TGFA | 0.451 | 0.101 | 0.17 | 0.000 |  | 0.501 | 0.109 | 0.20 | 0.000 |  | 0.151 | 0.344 | 0.02 | 0.661 |
| TNFB | -0.207 | 0.152 | -0.03 | 0.173 |  | -0.252 | 0.172 | -0.04 | 0.143 |  | 0.052 | 0.344 | 0.00 | 0.881 |
| TNFRSF9 | 0.168 | 0.139 | 0.02 | 0.227 |  | 0.160 | 0.156 | 0.04 | 0.303 |  | 0.256 | 0.334 | 0.01 | 0.444 |
| TNFSF14 | 0.299 | 0.114 | 0.06 | 0.009 |  | 0.383 | 0.139 | 0.06 | 0.006 |  | 0.127 | 0.214 | 0.03 | 0.551 |
| TRAIL | -0.006 | 0.156 | 0.00 | 0.968 |  | -0.024 | 0.178 | 0.00 | 0.893 |  | 0.164 | 0.335 | 0.01 | 0.625 |
| TRANCE | -0.106 | 0.101 | -0.02 | 0.294 |  | -0.133 | 0.118 | -0.04 | 0.260 |  | 0.066 | 0.207 | 0.01 | 0.750 |
| TWEAK | -0.013 | 0.167 | 0.00 | 0.936 |  | 0.046 | 0.184 | 0.00 | 0.804 |  | 0.028 | 0.434 | 0.00 | 0.948 |
| uPA | -0.100 | 0.208 | -0.03 | 0.631 |  | -0.124 | 0.241 | -0.01 | 0.607 |  | 0.062 | 0.426 | 0.00 | 0.884 |
| VEGFA | 0.114 | 0.124 | 0.01 | 0.357 |  | 0.115 | 0.133 | 0.01 | 0.386 |  | 0.242 | 0.418 | 0.01 | 0.563 |

**Supplementary Table 5. Results of the associations between inflammatory cytokines and ulcerative colitis using forward Mendelian randomization analyses.**

| Proteins | **Overall** | | | | |  | **Cis** | | | |  | **Trans** | | | |
| --- | --- | --- | --- | --- | --- | --- | --- | --- | --- | --- | --- | --- | --- | --- | --- |
|  | Cis/Trans | nSNP | OR (95% CI) | P value | P value (FDR) |  | nSNP | OR (95% CI) | P value | P value (FDR) |  | nSNP | OR (95% CI) | P value | P value (FDR) |
| CCL11 (Eotaxin) | Trans | 2 | 1.14 (1.09, 1.18) | 3.890E-10 | 4.280E-09 |  | - | - | - | - |  | 2 | 1.14 (1.09, 1.18) | 3.890E-10 | 3.110E-09 |
| CCL20 (MIP3A) | Trans | 1 | 0.98 (0.52, 1.82) | 0.939 | 0.939 |  | - | - | - | - |  | 1 | 0.98 (0.52, 1.82) | 0.939 | 0.939 |
| CCL4 (MIP1B) | Trans+cis | 2 | 1.14 (0.93, 1.39) | 0.223 | 0.488 |  | 1 | 1.02 (0.90, 1.17) | 0.723 | 0.868 |  | 1 | 1.26 (1.10, 1.44) | 0.001 | 0.004 |
| CXCL1 (GRO-a) | Cis | 1 | 0.98 (0.92, 1.04) | 0.486 | 0.594 |  | 1 | 0.98 (0.92, 1.04) | 0.486 | 0.729 |  | - | - | - | - |
| CXCL8 (IL-8) | Trans | 2 | 1.45 (0.75, 2.79) | 0.266 | 0.488 |  | - | - | - | - |  | 2 | 1.45 (0.75, 2.79) | 0.266 | 0.709 |
| CXCL9 (MIG) | Cis | 1 | 1.45 (1.08, 1.95) | 0.012 | 0.066 |  | 1 | 1.45 (1.08, 1.95) | 0.012 | 0.072 |  | - | - | - | - |
| HGF | Cis | 1 | 1.25 (0.97, 1.61) | 0.085 | 0.312 |  | 1 | 1.25 (0.97, 1.61) | 0.085 | 0.170 |  | - | - | - | - |
| IL-6 | Trans | 1 | 1.04 (0.81, 1.32) | 0.778 | 0.856 |  | - | - | - | - |  | 1 | 1.04 (0.81, 1.32) | 0.778 | 0.889 |
| MMP10 | Trans+cis | 2 | 0.89 (0.66, 1.20) | 0.452 | 0.594 |  | 1 | 0.82 (0.68, 0.98) | 0.025 | 0.075 |  | 1 | 1.15 (0.85, 1.56) | 0.357 | 0.709 |
| OSM | Trans | 1 | 1.09 (0.87, 1.36) | 0.443 | 0.594 |  | - | - | - | - |  | 1 | 1.09 (0.87, 1.36) | 0.443 | 0.709 |
| CASP8 | Trans+cis | 2 | 1.04 (1.03, 1.05) | 7.63E-19 | 1.22E-17 |  | 1 | 1.05 (0.76, 1.44) | 0.783 | 0.881 |  | 1 | 1.04 (0.83, 1.29) | 0.749 | 0.934 |
| ENRAGE | Trans | 1 | 1.00 (0.91, 1.10) | 0.948 | 0.948 |  | - | - | - | - |  | 1 | 1.00 (0.91, 1.10) | 0.948 | 0.948 |
| TNFSF14 | Trans+cis | 2 | 1.02 (0.99, 1.05) | 0.209 | 0.488 |  | 1 | 1.01 (0.89, 1.15) | 0.881 | 0.881 |  | 1 | 1.05 (0.85, 1.28) | 0.669 | 0.889 |

Figure S1. Stratified analysis of smoking status in the discovery cohort with adjustments of age, sex and Montreal classifications and study effect.

**Figure S2**. Stratified analysis of smoking status in the validation cohort with adjustments of age, sex and Montreal classifications and study effect.

**Figure S3.** Stratified analysis of smoking status in both cohorts with adjustments of age, sex and Montreal classifications and study effect.

**Figure S4.** Stratified analysis of Montreal classifications and study in the validation cohort with adjustments of age, sex and smoking effect.

**References**

1. Sun BB, Maranville JC, Peters JE*, et al.* Genomic atlas of the human plasma proteome. *Nature* 2018;**558**:73-9.

2. Folkersen L, Gustafsson S, Wang Q*, et al.* Genomic and drug target evaluation of 90 cardiovascular proteins in 30,931 individuals. *Nat Metab* 2020;**2**:1135-48.

3. Ahola-Olli AV, Würtz P, Havulinna AS*, et al.* Genome-wide association study identifies 27 loci influencing concentrations of circulating cytokines and growth factors. *American Journal of Human Genetics* 2017;**100**:40-50 %U <https://www.ncbi.nlm.nih.gov/pmc/articles/PMC5223028/>.

4. Ferkingstad E, Sulem P, Atlason BA*, et al.* Large-scale integration of the plasma proteome with genetics and disease. *Nat Genet* 2021;**53**:1712-21.

5. Liu JZ, van Sommeren S, Huang H*, et al.* Association analyses identify 38 susceptibility loci for inflammatory bowel disease and highlight shared genetic risk across populations. *Nature Genetics* 2015;**47**:979-86.
